# Detection of asymptomatic carriers of malaria in Kohat district of Pakistan

**DOI:** 10.1186/s12936-018-2191-y

**Published:** 2018-01-22

**Authors:** Muhammad Abdul Naeem, Suhaib Ahmed, Saleem Ahmed Khan

**Affiliations:** 1 Combined Military Hospital, Lahore, Pakistan; 2 Riphah International University, Islamabad, Pakistan; 3 Army Medical College, Rawalpindi, Pakistan

**Keywords:** Asymptomatic malaria, RT-PCR, Endpoint fluorometry

## Abstract

**Background:**

Kohat district is one of the medium intensity malaria transmission areas in Pakistan where asymptomatic carriers are likely to form a reservoir of infection. This study was done to explore the possibility of using microscopy, rapid diagnostic testing (RDT), real time polymerase chain reaction (RT-PCR) and RT-PCR followed by endpoint fluorometry (EPF) for detection of malaria in asymptomatic immediate family members of patients of malaria (homestead) and in a sample from the general population of Kohat.

**Methods:**

This cross-sectional study was done at Combined Military Hospital Kohat and Molecular Lab of Riphah International University, Islamabad from Jan to Dec 2015. A total of 1000 individuals including 200 microscopy positive patients of malaria, 400 asymptomatic immediate family members (homestead) of the active patients of malaria and 400 apparently healthy controls were tested by microscopy, RDT and RT-PCR. At the end of RT-PCR the result were read by EPF.

**Results:**

In the 200 malaria microscopy positive patients, 190 (95%) were RDT positive and all were RT-PCR positive. In the 400 individuals from the homestead of malaria patients, 6 (1.5%) individuals were malaria microscopy positive while RDT failed to pick any positive and 32 (8%) were RT-PCR positive for malaria. EPF of all the RT-PCR positive results were positive and the negative results were negative. The difference in the frequency of malaria in the homestead versus general population was very significant (*p* = 0.0002) and the relative risk of malaria was 4.0 times higher (95% CI 1.87–8.57).

**Conclusion:**

The chances of detecting asymptomatic malaria carriers is significantly higher in the homestead of malaria patients than in the general population and for this purpose RT-PCR with EPF can be very useful in the diagnosis of malaria especially with low parasite density.

**Electronic supplementary material:**

The online version of this article (10.1186/s12936-018-2191-y) contains supplementary material, which is available to authorized users.

## Background

Malaria is one of the common causes of death from infectious diseases, but remains the main global cause of death from parasitic infectious diseases [1]. Asymptomatic carriers of malaria provide a reservoir of infection in areas with low to medium intensity transmission that may contribute to continuous transmission of the disease and can ignite devastating epidemics [2]. Detection of the asymptomatic pool of carriers by easily accessible cheap and highly sensitive molecular diagnostic tools is necessary for successful eradication of malaria. Detection and treatment of the asymptomatic pool of carriers has been tried in a number of countries all over the world with varying degrees of success [3]. This concept has not gained popularity because of the high cost of molecular testing and limited accessibility to the end users because of logistic problems [4]. It has been observed that the immediate household of a positive patient of malaria experience higher than average exposure to infectious mosquitoes [5, 6]. A cost-effective approach for identification of asymptomatic carriers could be through screening of a group of selected individuals from the immediate household of an active patient of malaria. A sensitive RT-PCR based test for detection of malaria is the best method for this purpose [7]. Keeping in view the enormous potential of detecting asymptomatic individuals harbouring malaria by sensitive molecular methods this study describes a cost effective and highly sensitive molecular method for detection of low level parasitaemia in a targeted high risk population.

## Methods

This cross-sectional study was done at Combined Military Hospital (CMH) Kohat and Molecular Lab, Riphah International University, Islamabad from Jan to Dec 2015 to identify a suitable approach for detection of asymptomatic carriers of malaria in Kohat district of Pakistan. The study was approved by the ethical review committee of Riphah International University, Islamabad. A total of 1000 individuals were included in this study. This included 200 consecutive patients of malaria who presented to CMH Kohat and were confirmed on microscopy, 400 apparently asymptomatic individuals from the immediate family members (homestead) of active patients of malaria and 400 apparently healthy individuals randomly picked from the same resident community with similar socio-economic status. A written consent for participation was obtained from all of the study participants. Apparently healthy individuals not resident in the area for at least 2 years and those with history of fever in the last 2 months were excluded from the study.

Each individual was tested by microscopy for malaria, RDT, RT-PCR and EPF. Microscopy was done on Leishman stained thin smears. Each smear was examined by an experienced microscopist for 3–5 min. The slides of individuals who were negative on microscopy but were positive by RT-PCR were re-examined for at least 100 high power fields. The specie of malaria parasite was identified when visible on microscopy and the number of parasites per µL was calculated [8]. All microscopy or RT-PCR positive subjects of malaria were treated at CMH Rawalpindi.

Rapid diagnostic testing for malaria was done as per manufacturer’s instructions (SD BIOLINE Malaria Ag P.f/Pan product code 05FK60, Standard Diagnostics, Inc. Korea). It is a pan malaria RDT that can detect all species. DNA extraction was done from peripheral blood collected in EDTA by Chelex™ method (BioRad, USA). Red cells in 300 µL blood sample were lysed by distilled water. The process of red cell lysis was repeated more than once if any visible trace of haemoglobin was left behind. White cells including the malarial parasite were pelleted after centrifugation at 10,000 rpm for 2 min. The pellet was incubated at 95 °C for 20 min in 300 µL of 7% Chelex™. The clear supernatant was used as source of DNA.

PCR amplification was done by real time method targeting the genus *Plasmodium* specific 18S rRNA gene (SSUrRNA gene) [7, 9]. The target gene is shared by four malarial parasite species (*Plasmodium falciparum, Plasmodium vivax, Plasmodium malariae, Plasmodium ovale*). The following primers and TaqMan probe were used:

Forward: 5′-ACATGGCTATGACGGGTAACG-3′

Reverse: 5′-TGCCTTCCTTAGATGTGGTAGCTA-3′

TaqMan Probe: 6FAM 5′-TCAGGCTCCCTCTCCGGAATCGA-3′-BHQ1

DNA amplification was done in 20 µL reaction mixture containing 5 pM of each primer and the probe (IDT, USA), 0.5 units of Taq polymerase (Thermo Fisher Scientific, USA), 30 mM of each dNTP, 10 mM Tris HCl (pH 8.3), 50 mM KCL, 1.5 mM MgCl_2_, 100 mg/mL gelatine and 0.1–0.3 µg of genomic DNA (2 µL of extracted DNA). Real time PCR was done on Rotor-Gene 6000 (Corbett Research, Australia). Thermal cycling comprised initial denaturation at 95 °C for 2 min followed by 40 cycles of denaturation at 95 °C for 20 s and annealing/extension at 60 °C for 1 min. Green fluorescence was read at the 60 °C step.

The results of RT-PCR were recorded as cycle threshold (Ct). Samples with Ct ≤ 35 were taken as positive while all others were taken as negative. In each batch of PCR known positive and negative DNA for malaria were also included.

### Endpoint fluorescence (EPF)

At the end of RT-PCR the reaction vials of all positive and negative samples were read in a fluorometer (GTI PCR Reader, Genetic Technology Instrumentation, Pakistan, http://grcpk.com/gti-pcr-reader-g/). The instrument measures green fluorescence in 0.2 mL PCR reaction vials. The amount of fluorescence is recorded on a computer software and the results are expressed in relative fluorescence units (RFU) after subtracting background fluorescence of a known negative sample.

In order to validate the EPF results tenfold dilutions (1/1, 1/10, 1/100, 1/1000) of a RT-PCR positive DNA sample (*P. vivax* parasitaemia ~ 2000/µL) and one RT-PCR negative sample were run by the RT-PCR protocol. The dilutions and the negative sample were run under identical RT-PCR conditions in five separate reaction tubes each. At the end of RT-PCR EPF was measured as described earlier. The results of Ct values and the corresponding EPF of each dilution were cross tabulated. The cost of DNA extraction, RT-PCR and the technician time were calculated in US$ as per the local market price.

Data were analysed by SPSS version 22.0 for descriptive and inferential statistics. Frequency and percentages were calculated for positive and negative patients of malaria in the study groups. The frequency of occurrence in each group were compared by Chi square test. The relative risk of developing malaria in the individuals from the homestead of a patient with malaria was compared with that in the general population. Sensitivity and specificity of microscopy, RDT and RT-PCR followed by EPF for malaria were calculated while taking RT-PCR as the gold standard.

## Results

The results of microscopy, RDT, RT-PCR and RT-PCR-EPF in the three groups of subjects are summarized in Tables 1 and 2.

**Table 1:** Summary of microscopy, RDT, RT-PCR and RT-PCR-EPF for malaria in the three groups of individuals

Group	Microscopy		Rapid diagnostic testing		Real time PCR		RT-PCR EPF	
	Positive	Negative	Positive	Negative	Positive	Negative	Positive	Negative
Patients of malaria (200)	200 (100.0%)	0	190 (95.0%)	10 (5.0%)	200 (100.0%)	0	200 (100.0%)	0
Asymptomatic homestead (400)	6 (1.5%)	394 (98.5%)	0	400 (100%)	32 (8.0%)	368 (92.0%)	32 (8.0%)	368 (92.0%)
Healthy controls (400)	0	400 (100%)	0	400 (100%)	8 (2.0%)	392 (98.0%)	8 (2.0%)	392 (98.0%)

**Table 2:** Results of microscopy, RDT, RT-PCR and RT-PCR-EPF in 240 patients positive for malaria

Patients (240)	15	35	52	85	53
Parasitemia	< 50/µL	50–100/µL	100–1000/µL	1000–10,000/µL	> 10,000/µL
Microscopy	−	±	+	++	+++
RDT	−	−	+	++	+++
RT-PCR (Ct)	31.9 (30.0–34.0)	28.6 (27.0–29.9)	25.4 (24.0–26.9)	22.5 (21.0–23.9)	19.6 (16.0–20.9)
RT-PCR-EPF (RFU)	51 (27–81)	89 (48–137)	142 (105–181)	182 (109–254)	227 (178–289)

### Microscopy

In the 200 microscopy positive patients 196 (98%) had *P. vivax* infection whereas the remaining four had *P. falciparum.* The parasite density ranged from 100/µL to > 10,000/µL. In the 400 individuals from the homestead group microscopy revealed malaria parasite in 6 (1.5%) individuals. All of the six had *P. vivax* malaria. The parasite density ranged from 70 to 500/µL. Microscopy did not show malarial parasite in any of the 400 apparently healthy controls.

### RDT

In the 200 lab detected patients of malaria 190 (95%) were positive on RDT. RDT failed to pick any positive case out of the 800 subjects from the homestead and the population controls.

### RT-PCR

All of the microscopy positive patients of malaria were positive on RT-PCR. In the 400 subjects from the homestead group 32 (8%) were positive for malaria (Table 1) whereas in the 400 healthy controls 8 (2%) were positive on RT-PCR (Table 1). There was a good correlation between the parasite density and the RT-PCR results (Table 2). All of the patients that could not be picked by microscopy were weak positive on RT-PCR (Ct 30.0–34.0). The patients that were easily picked by microscopy or RDT had Ct below 27.0.

### Endpoint fluorometry (EPF)

The results of EPF validation assay showed good correlation between the RT-PCR and the EPF results (r = − 0.9853; see Additional file 1). The EPF was as sensitive as RT-PCR with the lowest detection limit of ~ 1–2 parasites/µL. Keeping in view the mean EPF result in the negative samples (4 RFU, range 0–11) and the highest dilution of positive DNA (~ 2 parasites/µL) (31 RFU, range 21–42) the cut off limit for positive was arbitrarily defined at 20 RFU.

**Fig. 1:**
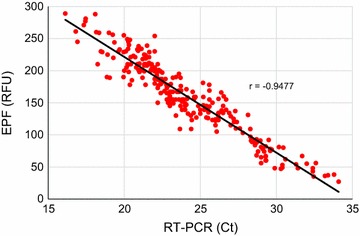
Correlation between RT-PCR cycle threshold (Ct) and the corresponding EPF relative fluorescence units (RFU) in 240 samples positive for malaria

The EPF results in 240 RT-PCR positive patients ranged from 27 to 289 RFU (Table 2). The results of EPF correlated well with RT-PCR (r = − 0.9477) (Fig. 1). In eight RT-PCR negative samples EPF had discordant result (22–36 RFU). However, all of these samples gave clear negative result on re-extraction of DNA. The RT-PCR negative samples had mean EPF value of 5 RFU (range 0–15). EPF did not give any false positive or false negative result.

Microscopy and RDT were as sensitive as RT-PCR in patients with parasite density above 100/µL (Table 3). However, in the subjects with parasite density below 100/µL (most of the asymptomatic carriers) microscopy was only 15% sensitive (95% CI 6–30%) and RDT was not sensitive (95% CI 0–9%). The specificity of microscopy, RDT, RT-PCR and EPF was 100% (95% CI 99–100%). The cost of DNA extraction, RT-PCR and the technician time was estimated at US$ 4.0 per test.

**Table 3:** Sensitivity and specificity of microscopy, RDT and RT-PCR-EPF in the 800 asymptomatic individuals tested for malaria

	Microscopy	RDT	RT-PCR-EPF
Sensitivity	15% (6–30%)	0% (0–9%)	100% (91–100%)
Specificity	100 (99–100%)	100% (99–100%)	100% (99–100%)

RT-PCR was taken as gold standard

There was a very significant difference between the frequency of individuals carrying malarial parasite in the homestead of active patients of malaria (8%) and the general population (2%) (*p* = 0.0002). The relative risk of malaria in the household of an index patient was 4.0 times higher than in the general population (95% CI 1.87 to 8.57).

## Discussion

Malaria is the leading cause of morbidity and mortality due to vector born disease in Pakistan [10]. An estimated one million and confirmed 300,000 cases of malaria occur each year in Pakistan [11]. The reported annual parasite incidence indicate that Khyber Pakhtunkhwa, Federally Administered Tribal Areas (FATA) and Baluchistan are the highest endemic provinces in Pakistan. Around 80% of malaria cases reported in Pakistan are due to *P. vivax* while the remaining 20% are caused by *P. falciparum*. The cumulative overall transmission rate of malaria in the Khyber Pakhtunkhwa province is 4.3/1000 population [12].

In geographical areas with intense transmission of malaria the population develops high level of immunity and malaria remains “stable” with no epidemics. On the other hand when the malaria transmission is intermittent due to seasonal variations the infection is “unstable”. In such populations the level of immunity is low, therefore, epidemics are common and the disease may also run a severe course [2]. Malaria transmission in Pakistan is mostly seasonal and of unstable pattern [10]. However, intense transmission occurs in the bordering regions with Iran and Afghanistan and along coastal belt in Sindh and Baluchistan [11]. In these areas, a large number of individuals are expected to have high immunity to malaria and many of them may harbour low level parasitemia without having symptoms [2]. The asymptomatic carriers of malaria become a reservoir of infection and may become an obstacle in the success of malaria eradication [3].

District Kohat is located in Khyber Pakhtunkhwa and WHO has defined it as a region of low to moderate transmission setting where the annual malaria transmission rate is < 10%/1000 population [12]. It is expected that large number of asymptomatic carriers of malaria would be present in this area [2]. The results of the current study also conform to this expectation. The asymptomatic carriers act as reservoir of malaria from where the mosquitoes continue to feed and spread the parasite to other people. The residual source of transmission may remain undetected unless sensitive methods of detection like PCR are employed [13]. Residual malaria transmission can persist after achieving full universal coverage with effective insecticidal nets and/or indoor spraying with ingredients to which local vector populations are fully susceptible [3]. The asymptomatic carriers may have an important contribution in residual transmission of malaria [2].

In this study RDTs failed to detect low level parasitaemia in both symptomatic and asymptomatic patients of malaria. Similarly, thin film microscopy also lacked the sensitivity to pick low level parasitaemia. Similar observations have also been reported in an earlier study from Pakistan [14]. Microscopy and RDT frequently missed detection of low level parasitaemia in asymptomatic malaria patients in Myanmar, Nigeria, Venezuela, Iran, Tanzania, Kenya, India, and Sudan [15–23]. Microscopy for malaria is a subjective assessment tool that may be influenced by examiner’s expertise and the time spent on examination [24]. This study clearly shows that most of the asymptomatic carriers have low level parasitaemia (below 100/µL) and these individuals can be easily missed by microscopy or RDTs. The best method to identify them would be to use sensitive molecular methods. The good correlation between quantitative PCR and the level of parasitaemia, seen in this study, has also been reported previously [25].

Detection of low-density parasite count by PCR in asymptomatic patients has far-reaching implications for areas with moderate transmission in Pakistan. It is important that the asymptomatic patients are identified and treated otherwise malaria control cannot be successful in these areas of Pakistan. Mathematical models frequently predict erroneous prevalence rates based on detection of malaria in asymptomatic individuals by routine slide examination. Strategies solely relying on microscopy and RDT results can end up in failure. This study augments the reason to use PCR for accurate estimates of the infectivity levels of the sub microscopic reservoir in the asymptomatic individuals [26]. Major hurdle in the use of PCR in detection of asymptomatic patients of malaria in countries with resource constraints is the high cost and the difficulties in establishing PCR laboratories in remote areas [27]. Loop mediated isothermal amplification (LAMP) is a low cost sensitive molecular method for diagnosis of malaria that can be performed in routine labs because it does not require thermal cycler [28]. The EPF method could not be compared with the LAMP method because the commercial LAMP kits are not available in Pakistan. The EPF method is cheap (~ US$ 4.0 per test) as well as sensitive with detection limit around 2 parasites/µL. The EPF equipment (GTI PCR reader) costs around US$ 1000. It is a user friendly device with sensitivity comparable to RT-PCR. The instrument is primarily meant for the qualitative testing but its results also give a good correlation with the quantitative RT-PCR results. Besides the instrument cost the EPF reading incurs no extra cost. The EPF initially gave very weak false positive results in eight individuals. On re-extraction of the DNA all of these results were clear negative. The false positivity appears to be due to the fluorescence emitted by traces of haemoglobin leftover form the extraction. For EPF applications it is important to completely remove haemoglobin form the DNA samples. The EPF method can also provide rough estimate of parasitaemia with sensitivity to detect one to two parasites per microlitre. The EPF method has a clear edge over microscopy and RDT in detection of asymptomatic individuals with low level of parasitaemia. In the EPF method RT-PCR machine can be replaced with any conventional thermal cycler.

## Conclusion

The frequency of asymptomatic carriers of malaria is significantly higher in the homestead of patients of malaria than in the general population. Microscopy and RDT are not suitable for their detection. They are best detected by RT-PCR or PCR with EPF.

## Additional file

**Additional file 1: Figure S1.** Correlation between RT-PCR cycle threshold (Ct) and the corresponding EPF relative fluorescence units (RFU) in tenfold diluted malaria positive sample. **Table S1.** Results of RT-PCR (Ct) and EPF (RFU) in tenfold diluted DNA sample positive for malaria and a negative DNA.
