# Supplementary material for: Detection of asymptomatic carriers of malaria in Kohat district of Pakistan
Source: Malar J. 2018 Jan 22;17:44. doi: 10.1186/s12936-018-2191-y (PMC5778661; doi:10.1186/s12936-018-2191-y)
Supplement: Supplementary file 1 — Additional file 1: Figure S1. Correlation between RT-PCR cycle threshold (Ct) and the corresponding EPF relative fluorescence units (RFU) in tenfold diluted malaria positive sample. Table S1. Results of RT-PCR (Ct) and EPF (RFU) in tenfold diluted DNA sample positive for malaria and a negative DNA. [file 12936_2018_2191_MOESM1_ESM.docx]

Table S1. Results of RT-PCR (Ct) and EPF (RFU) in tenfold diluted DNA sample positive for malaria and a negative DNA.

| Dilution (X5) | 1/1 | 1/10 | 1/100 | 1/1000 | Negative |
| --- | --- | --- | --- | --- | --- |
| Parasitaemia | 2000/µL | 200/µL | 20/µL | 2/µL | 0/ µL |
| RT-PCR (Ct) | 21.2 (21.0-21.5) | 25.0 (24.7-25.2) | 28.8 (28.6-29.2) | 35.3 (33.8-37.3) | - |
| EPF (RFU) | 162 (150-176) | 140 (138-151) | 94 (77-104) | 30 (21-42) | 4 (0-11) |

Fig S1. Correlation between RT-PCR cycle threshold (Ct) and the corresponding EPF relative fluorescence units (RFU) in tenfold diluted malaria positive sample.
